# Job adjustment predictive factors of healthcare midwives in health system reform in Iran

**DOI:** 10.1186/s13690-023-01193-1

**Published:** 2023-10-10

**Authors:** Monireh Rezaee Moradali, Sepideh Hajian, Hamid Alavi Majd, Mohammadreza Rahbar, Rasool Entezarmahdi

**Affiliations:** 1 Student Research Committee, School of Nursing and Midwifery, Shahid Beheshti University of Medical Sciences, Tehran, Iran; 2 Department of Midwifery, School of Nursing and Midwifery, Shahid Beheshti University of Medical Sciences, Vali Asr Ave., Niayesh Cross Road, Niayesh Complex, Tehran, 1996835119 Iran; 3 Department of Biostatistics, School of Allied Medical Sciences, Shahid Beheshti University of Medical Sciences, Tehran, Iran; 4 Skin and Stem Cell Research Center, Tehran University of Medical Sciences, Tehran, Iran; 5 Assistant Professor of Epidemiology, Social Determinant of Health Research Center, Clinical Research Institute, Biostatistics & Epidemiology Department, Medical School, Urmia University of Medical Sciences, Urmia, Iran

**Keywords:** Adjustment, Job satisfaction, Work engagement, Midwife, Health care reform

## Abstract

**Background:**

Possessing sensitive and multiple responsibilities in the country's health system, particularly after the implementation of the health reform in Iran, midwives must be able to optimally perform their duties in their new job as healthcare providers. This study aimed to identify the factors that predict job adjustment for Iranian midwives working in healthcare.

**Methods:**

In this cross-sectional study, 310 midwives were recruited from 209 health centers in the Iranian province of West Azerbaijan using the census method and asked to complete research questionnaires. Data were collected using job adjustment, job satisfaction, and organizational commitment scales. SPSS version 25 was used to perform ANOVA and calculate multiple linear regression coefficients for data analysis. In addition, the AMOS software was employed for path analysis and the identification of predictive variables.

**Results:**

The mean age of the participants was 37.67 ± 7.1 years. Most participants (35.5%) were interested in their occupation as a midwife, and 27.1% were very interest. They had a moderate to strong tendency (76.1%) to remain in their new profession. In addition, 58.1% of participants experienced moderate job adjustment. For healthcare midwives, "desire to remain in the midwifery profession" and "organizational commitment" were significant predictors of job adjustment. "Desire to remain in the midwifery profession" directly affected midwives' job adjustment, while "interest in the new profession" had an indirect effect. Furthermore, "adequacy of income to expenses," "job satisfaction," and "organizational commitment" through the mediating role of "desire to remain in the profession" can, directly and indirectly, influence their job adjustment.

**Conclusion:**

To better prepare midwives for their role as healthcare providers, organizational managers should focus their efforts and plan primarily on providing incentives to increase the longevity of staying in the profession of midwifery increase job adjustment, job satisfaction, and organizational commitment, thereby improving the quality-of-service delivery.

**Table:** 

Text box 1. Contributions to the literature
• Considering the average level of adaptation of health care midwives with their current professional position, this problem requires targeted planning by organizational managers.
• Improving the quality of midwifery services requires increasing job satisfaction and organizational commitment in order to improve midwives' compatibility with the role of health care providers.
• Financial incentives, modification of multidimensional tasks and motivational programs are effective in increasing the desire of midwives to stay in the health care profession, as one of the important predictors of job adaptation.

## Introduction

Health is one of the most important pillars of development in any country, and the health system plays a crucial role in maintaining and improving the various dimensions of people's health; therefore, health justice is a key requirement of every society [1–3]. In this regard, one of the strategies involves using a referral system and implementing primary healthcare strategies by employing educated personnel to provide equitable services, particularly to the population of girls and mothers [4, 5].

Reform of Iran's health system began in 2013 with three goals: financial protection of patients, equitable access to health services, and service quality enhancement [6, 7]. As a significant factor in health care reform, employee satisfaction can be considered a determinant of service provision quantity and quality [8, 9]. Greater attachment to the organization, a heightened sense of responsibility, and organizational commitment result from a sense of job satisfaction and job security [10].

Today, improving the quality of midwifery services is one of the most contentious international issues. Midwives play significant roles in maintaining and improving the health of mothers and children at three levels of care: health and treatment facilities, family, and society [11]. Midwives in developing countries face numerous challenges in the areas of education, laws, lack of autonomous professional associations, and management and support issues [12].

The most fundamental problem is midwives' unclear identity and job responsibilities, which can, due to factors such as a lack of job planning and inappropriate service conditions, including salaries, rewards, and educational benefits, not only impede their professional advancement but also lead to job burnout due to working in uncertain conditions, an insecure workplace, and the absence of an appropriate referral system [12]. In contrast, many developed countries provide opportunities for midwives to strengthen their skills in organizing and directing local services for women and families by formulating community-based policies [13].

According to Herzberg's theory of two factors, there are two categories of needs. The first category, health factors, refers to circumstances where their absence causes dissatisfaction, but their presence does not generate intense and powerful motivation. The second category consists of motivational factors that lead to motivation and satisfaction in individuals whose absence produces only moderate levels of dissatisfaction. In general, motivational factors are the primary cause of job satisfaction, whereas the absence of health factors is the primary cause of job dissatisfaction [14].

Job adjustment refers to the behaviors that lead to good task performance, a positive attitude toward a new job, and psychological compatibility with the job after being hired [15, 16]. Dawis and Lofquist found a significant correlation between job satisfaction and job adjustment. If a person's skills are compatible with their job conditions, they will be satisfied and remain in that position [17].

While reducing conflicts and contradictions and adjusting one's job can improve work efficiency [16] and performance [18] and reduce job tensions [19] and mental disorders [20]. After promptly identifying the barriers to job adjustment and the needs of employees in the workplace, the necessary grounds for establishing job adjustment and satisfaction and formulating future plans in this regard can be provided.

Moreover, several studies have demonstrated that job satisfaction and adjustment are, to some extent, influenced by an individual's commitment to the organization [21]. In two studies on the personnel of hospitals and health centers affiliated with the Alborz and Ahvaz Universities of medical sciences in Iran, researchers observed that job satisfaction has a direct and significant effect on organizational commitment, such that increased job satisfaction made the personnel feel more accountable for their organizational structure [22]. In addition, another study of social security organization employees in the province of Semnan found no direct relationship between job adjustment and organizational commitment; however, these two factors had a significant indirect effect on each other via the mediating role of job satisfaction and organizational trust [23].

Organizational commitment is a type of psychological dependence on the organization and refers to the relationship between the individual and the organization; it also plays a significant role in explaining the individual's behaviors and attitudes [21]. According to Buchanan (1974), organizational commitment is demonstrated by the three main parameters of emotional commitment, normative commitment, and continuance commitment in an individual's attitude toward the organization and is a tool for internalizing the organization's goals and values [24]. High organizational commitment makes it easier for organizations to achieve their goals and objectives and reduces absenteeism and time away from work [25]. However, it is unclear which factors are the foundations of job adjustment, whether they have a synergistic effect, or which factor is a prerequisite for another.

The recruitment of midwives as physician assistants in the urban family physician program has been one of these challenging aspects of Iran's healthcare system reform. This position was later renamed "healthcare midwife," and midwives had to adjust to the new title. Unemployment among midwives is one of the factors that can impact their job adjustment [26]. However, this name change makes a midwife a multi-professional who, in addition to the professional duties outlined in the midwifery academic education program, must shoulder the burden of several additional responsibilities [27].

In addition to performing their duties, healthcare midwives engage in non-midwifery activities. These activities consist of identifying the geographical environment where they provide services and the population they serve, creating an electronic health record and providing services to all age groups of this population, individual and family counseling, screening of the at-risk population, follow-up and care of patients and referral to higher levels, registration and reporting of cases, nutritional activities, school hygiene and mental health, screening of middle-aged and older men and women for diabetes and blood pressure, and follow-up of at-risk people and their referral [26].

According to several Iranian studies, this reform has increased the number of midwives leaving the field for various reasons, including poor educational and employment conditions, environmental factors, and a sense of instability and low job security [28]. Moreover, financial, cultural, educational, motivational, structural, programming, and executive problems, as well as fundamental problems such as unclear working hours, increased workload, multiple, unclear, and heavy tasks, inadequate instruction of personnel, and payment delays, are cited as problems of healthcare midwives [29].

About 66,394 experts are working on the plan to reform Iran's health system. In the province of West Azerbaijan, approximately 1,155 experts (midwives, healthcare providers, healthcare midwives) are working on the health system reform plan, 579 of whom have midwifery training, and 362 are healthcare midwives [30]. Most of the 209 comprehensive health service centers and health centers lack a midwife or healthcare midwife occupational rank [30]. In this province, family midwives work in villages in positions and jobs that are sometimes unrelated to their academic training. Despite providing midwifery services to high-risk pregnant mothers and juggling an excessive number of assigned tasks in a population with a high degree of ethnic and cultural diversity, they lack a defined job rank and status within the healthcare industry.

Based on the background above, the present research aimed to investigate the adjustment status of midwives to their new professional role as healthcare provider midwives in the province of West Azarbaijan through a cross-sectional-analytical study and to identify and analyze the most significant direct and indirect predictive factors in one's adjustment to the role of healthcare provider. Preventing, controlling, and reducing the stressful factors in the workplace and promoting positive adjustment methods, the results of this study are anticipated to facilitate the creation of the grounds for increasing the satisfaction of healthcare midwives with their duties and the quality of health services provided to the recipients of these services.

## Methods

This cross-sectional-analytical study was conducted in West Azarbaijan province in 2022. The research setting consisted of 209 health service centers, including 50 comprehensive urban health service centers, 45 comprehensive urban and rural health service centers, and 114 healthcare units covered by the main health centers, where healthcare midwives provided medical care. First, after receiving their written consent, all 362 midwives working as healthcare midwives in these centers were included in the study using the census method. The required information was then gathered by sending the electronic link to the study questionnaires to the participants' mobile phones.

Inclusion criteria included at least one year of experience working in health centers, residence in the province of West Azarbaijan, possession of a midwifery-related academic degree, acceptance of the electronic consent form to participate in the study, and possession of a smart mobile phone. Those who did not meet the criteria above or respond to any of the questionnaire questions were excluded from the statistical analysis.

The following questionnaires were used for the research: a demographic information questionnaire, a survey of some occupational characteristics, a socioeconomic status questionnaire with six demographic questions and five main questions on a 5-point Likert scoring scale [31], a job adjustment questionnaire developed by Dawis and Lofquist with 36 items [32], a job satisfaction scale developed by Herzberg with 40 items [33, 34], and Allen and Meyer's organizational commitment scale with 24 items and a 7-point Likert scoring scale [35]. Various Iranian studies previously confirmed the psychometrics of each of these scales [34, 36, 37].

As the target population for the selected tools was different in this study, their reliability was determined again using the test re-test method on 20 healthcare midwives outside of the study's final population with a two-week interval, and the intra-class correlation coefficient (ICC) was calculated by visiting and completing the questionnaires in person. In this regard, the reliability of the questionnaires was as follows: job adjustment questionnaire (0.893), job satisfaction questionnaire (0.997), organizational commitment questionnaire (0.994), and socioeconomic questionnaire (0.896).

The data were analyzed in SPSS version 25 using parametric or non-parametric equivalent tests based on the study variables and the data distribution's normality. Descriptive and inferential statistics were used to test each research hypothesis, including frequency calculation, mean, independent samples t-test, Pearson and Spearman correlation, and one-way variance analysis (ANOVA). The significance level was considered to be lower than 0.05. Moreover, using multiple linear regression and path analysis, AMOS software was used to determine predictive factors.

We constructed initial path models in AMOS with mediating variables using structural equation modeling (SEM). The following values were used to evaluate the indices for SEM analysis: The ratio of the chi-square (x^2^) statistic to its degree of freedom; a value less than 5 indicates an acceptable fit. Several fit indices were recommended after intense scrutiny to evaluate model fit. These included the root mean square error of approximation (RMSEA), the adjusted goodness-of-fit index (AGFI), the goodness-of-fit index (GFI), and the comparative fit index (CFI). The model was considered to be a good fit if the RMSEA ≤ 0.08, AGFI > 0.80 and other indices (CFI, GFI) were more than 0.90 [38, 39].

Given the central limit theorem and the sample size of 310 participants, it was assumed that the distribution of this variable is normal. Consequently, it served as the response variable in the multiple linear regression model.

In addition to the factors that directly affect job adjustment, such as job satisfaction, desire to persist, and organizational commitment, other factors, such as adequate income, can indirectly affect job adjustment, according to the literature review. In addition, the regression model cannot evaluate indirect effects; thus, path analysis was used to achieve this objective.

A path analysis diagram, as shown in Fig. 3, depicts the direct and indirect effects of factors affecting job adjustment. In addition, the standardized and non-standardized coefficients and *p*-values are presented in Table 6 for greater clarity regarding the results. In addition, Table 7 provides the exact values of the direct and indirect effects of the investigated variables. Table 5 displays the results of evaluating the data's compatibility with the model (goodness of fit) using necessary and common indicators in this field. As can be seen, the values of these indicators indicate that the path analysis model fits the data perfectly.

## Results

There were 362 links sent to healthcare midwives to complete a questionnaire; 323 were completed, while the remaining 39 were not. Exclusions were made from the primary study because three participants had less than a year of work experience, and ten participants did not answer all of the questions correctly on the questionnaires. In the end, 310 surveys' worth of data were analyzed.

The participants' mean age was 37.67 (7.1) years, their mean length of service was 10.26 (6.91) years, and they worked 43.20 h per week. In addition, approximately 84.6% of midwives were married, the majority had one or two children, 69% held a bachelor's degree, 93.2% worked in their native residences, and the majority owned their own homes. Furthermore, 40% of the participants were employed in healthcare units, and 44.5% held full-time contractual positions. Furthermore, 76.1% held the organizational position of health care midwife, and approximately 40.6% had previously worked as midwives in health centers. Most were highly interest with their new profession as midwives and desired to remain in it for a long time (Table 1).

**Table 1:** Absolute and relative frequency of demographic and occupational characteristics of healthcare midwives participating in the study in West Azarbaijan province in 2022 (*n* = 310)

**Variable**		**Frequency**	**Percent**
Marital status	Married	253	81.6
	Single	45	14.5
	Divorced	12	3.9
	TOTAL	310	100
Academic degree	Associate degree of midwifery	77	24.8
	Bachelor’s degree of midwifery	214	69.1
	Master’s degree of midwifery	11	3.5
	Related master's degree	8	2.6
	TOTAL	310	100
Adequacy of income to expenses	Very inadequate	52	16.8
	Inadequate	128	41.3
	Partly adequate	103	33.2
	Adequate	26	8.4
	Very adequate	1	0.3
	TOTAL	310	100
Economic status	Very low	9	2.9
	Low	60	19.4
	Average	226	72.9
	High	14	4.5
	Very high	1	0.3
	TOTAL	310	100
Personal housing price evaluation (Home owner)	Very low	13	5.6
	Low	38	16.3
	Average	148	63.5
	High	28	12.0
	Very high	6	2.6
	TOTAL	233	100
Ability to buy a house (No personal housing)	Very low	50	64.9
	low	23	29.9
	Average	2	2.6
	High and very high	2	2.6
	TOTAL	77	100
Place of occupation	Urban health service centers	93	30
	Urban–rural health service centers	93	30
	Healthcare unit	124	40
	TOTAL	310	100
Employment status	Full- time Contractual	138	44.5
	Full- time formal	109	35.2
	Part time	63	20.3
	TOTAL	310	100
Interest in job	Not interested	13	4.2
	Very low interest	18	5.8
	Low interest	7	2.3
	Medium interest	78	25.2
	High interest	110	35.5
	Very high interest	84	27.1
	TOTAL	310	100
Desire to stay in the profession	No desire	22	7.1
	Very low desire	27	8.7
	Low desire	25	8.1
	Medium desire	79	25.5
	High desire	77	24.8
	Very high desire	80	25.8
	TOTAL	310	100

Regarding socioeconomic factors, approximately 41.3% of respondents felt their income was insufficient to cover their living expenses, while 73.9% described their economic status as average. Among those who did not own their own homes, 64.9% stated they could not afford one (Table 1).

According to the findings of the primary analysis, 58.1% of the participants experienced moderate job adjustment. The mean raw score for job adjustment was 49.81 on a scale from 0 to 100. The values of altruism and autonomy were related to the highest and lowest levels of job adjustment, respectively (Table 2).

**Table 2:** Job adjustment and its domains in healthcare midwives participating in the study in West Azarbaijan province in 2022 (*n* = 310)

Job adjustment		Frequency (percentage)			
Factor	Proportion	Low	Average	High	
	Score	36–72	73–108	109–180	
	Frequency	4 (1.3%)	180 (58.1%)	126 (40.6%)	
	Minimum	Maximum		Domain score Mean (SD)	Score based on 0 to 100^a^ Mean (SD)
Total job adjustment (36 items)	40	150		106.25 (14.44)	49.81 (10.81)
Achievement value (3 items)	3	15		9.89 (2.47)	57.39 (20.64)
Comfort value (13 items)	13	55		34.94 (6.13)	52.22 (14.59)
Status value (4 items)	4	19		10.73 (3.31)	44.83 (22.12)
Altruism value (5 items)	5	24		18.05 (2.83)	68.70 (14.89)
Safety value (6 items)	6	27		18.86 (2.93)	61.29 (13.76)
Autonomy value (2 items)	2	10		4.64 (1.85)	33.02 (23.16)
Adjustment style (3 items)	3	15		9.14 (2.37)	51.18 (19.81)

^a^For the convenience of comparing the total scores and the scores of the areas after calculating the scores, they have been converted from zero to 100

Based on the study results, "external factors" were the most influential domain in determining job satisfaction for the participants (health factors). Salary and job security sub-structures had the greatest impact on job satisfaction, while workplace conditions sub-structure had the least impact. From the participants' perspectives, the sub-structures of job responsibility and work itself and achievement received the highest and lowest scores, respectively, among the "internal factors" (motivational factors). Moreover, most healthcare midwives (55.8%) demonstrated a strong organizational commitment. Emotional and normative commitment domains exhibited the highest and lowest organizational commitment levels, respectively (Table 3).

**Table 3:** The level of factors affecting job satisfaction and organizational commitment and their domains in healthcare midwives participating in the study in West Azarbaijan province in 2022 (*n* = 310)

Factor	Job satisfaction Frequency (percentage)						
	Scale	Weak		Average		Strong	
	Score	40–67		68–100		101–160	
	Frequency	10 (2.3%)		74 (23.9%)		226 (72.9%)	
	Domain	min/Max		Domain score Mean (SD)		Score based on 0 to 100 Mean (SD)	
Total job satisfaction		40/160		119.68 (28.51)		79.74 (20.99)	
External factors (health)	Salary/benefits	3/12		9.67 (2.87)		83.22 (30.11)	
	Company policy	3/12		9.07 (2.63)		81.20 (29.21)	
	Coworker relations	5/20		15.98 (3.56)		82.12 (29.52)	
	Job security	4/16		12.44 (3.41)		83.33 (24.54)	
	Work conditions	3/12		8.94 (3.00)		76.6 (32.20)	
	Supervision	5/20		15.02 (4.12)		82.41 (24.22)	
	Total score	23/92		71.12 (16.58)		81.48 (22.52)	
Internal factors (motivational)	Recognition	5/20		13.93 (4.68)		76.16 (26.93)	
	Advancement	4/16		11.09 (3.85)		78.01 (24.77)	
	Work itself	3/12		9.28 (2.38)		75.59 (35.21)	
	responsibility	3/12		8.63(2.82)		82.90(21.94)	
	Achievement	2/8		6.35(1.97)		75.59(29.90)	
	Total score	17/68		48.56(13.76)		77.65(23.07)	
Factor	Organizational commitment Frequency (percentage)						
	Scale		Very low	Low	High		Very high
	Score		24–59	60–95	96–131		132–168
	Frequency		7 (2.3%)	113 (36.5%)	173 (55.8%)		17 (5.5%)
	Min	Max	Domain score Mean (SD)		Score based on 0 to 100 Mean (SD)		
Total Organizational Commitment	40	154	100.61(20.62)		53.16(18.09)		
Emotional commitment	8	56	34.52(10.59)		55.24(22.07)		
Continuance commitment	15	51	33.90(5.48)		52.50(15.22)		
Normative commitment	8	56	32.10(8.53)		50.21(17.77)		

The mean job satisfaction score of healthcare midwives was 106.25 (14.44), representing a moderate level. Regarding job satisfaction, they received a mean score of 119.68 (28.51), and 72% of respondents believed that all the items introduced in this domain were essential for achieving job satisfaction. In addition, the study participants' mean organizational commitment score was 100.61 (20.62), indicating a high level of organizational commitment.

**Fig. 1:**
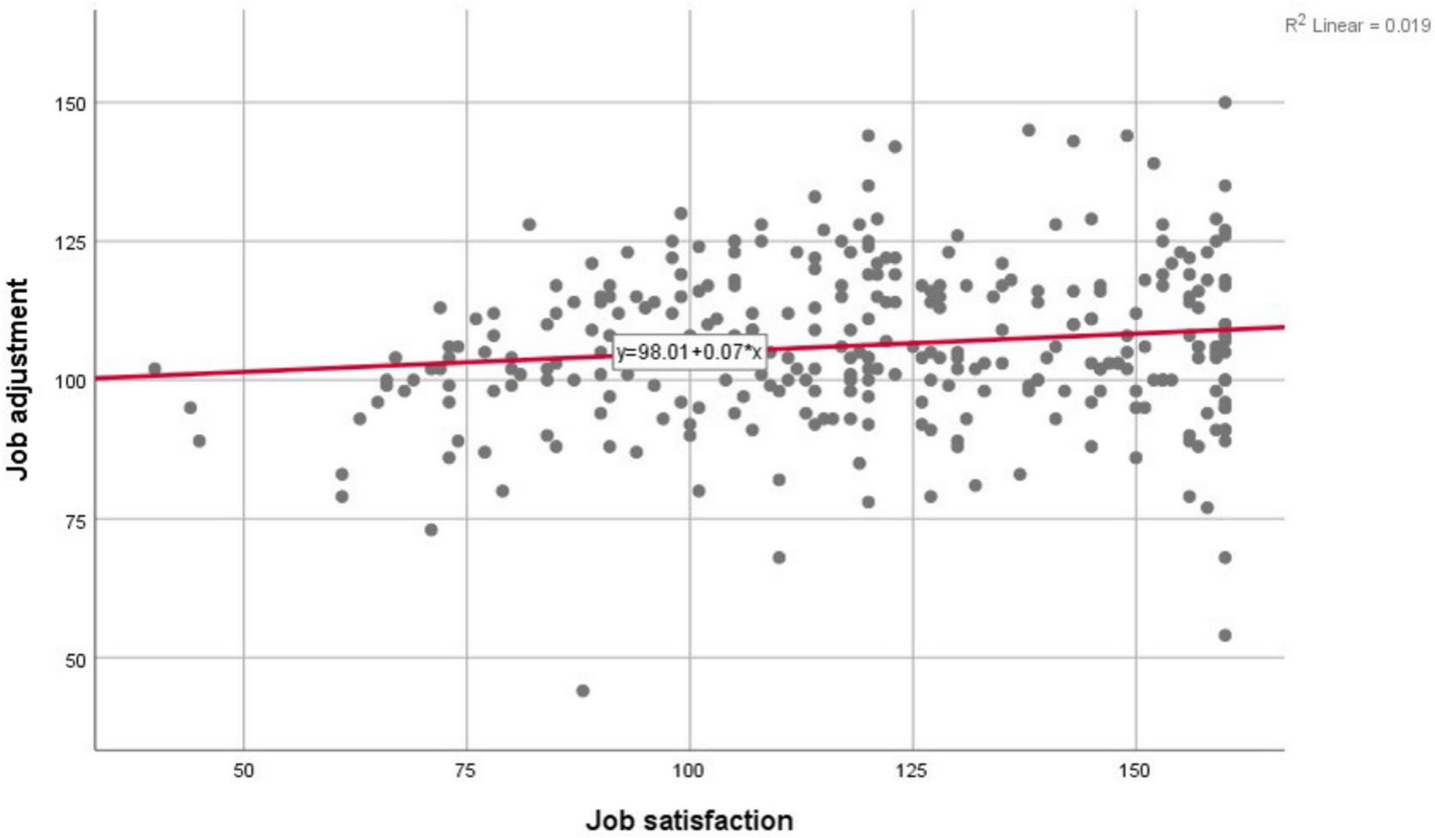
Scatter plot of highly correlated residuals in the regression model of job adjustment with job satisfaction in healthcare midwives participating in the study in West Azarbaijan province in 2022 (*n* = 310)

**Fig. 2:**
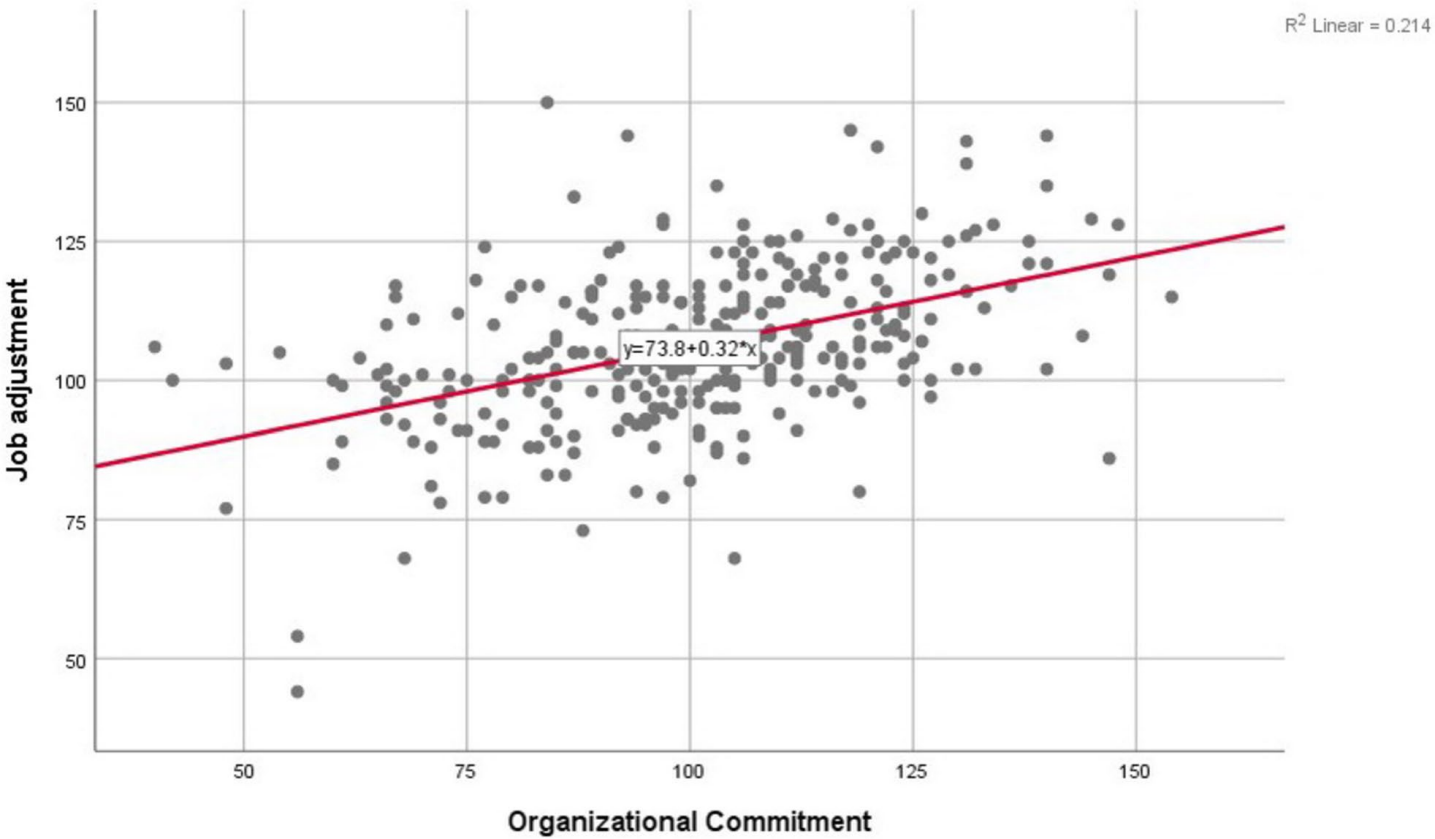
Scatter plot of highly correlated residuals in the regression model of job adjustment with organizational commitment in healthcare midwives participating in the study in West Azarbaijan province in 2022 (*n* = 310)

According to the results of Pearson's test, job adjustment was significantly correlated with age, population covered, job satisfaction, and organizational commitment (*p* < 0.05). In addition, based on the results of Spearman's test, job adjustment was significantly correlated with the variables of desire to remain in the profession, interest in the profession, adequate income, economic class, and housing price evaluation (*p* < 0.05) (Figs. 1 and 2).

Furthermore, a one-way ANOVA and independent t-test revealed that workplace structure (*p* < 0.001), employment status (*p* = 0.001), organizational position (*p* = 0.002), and previous job type (*p* = 0.05) had a significant impact on job adjustment (*p* < 0.05). This difference, however, was not statistically significant for the variables of marital status, housing status, and employment in the city of residence (*p* < 0.05).

Multiple regression (the enter method) was used to investigate the simultaneous effect of several significant individual, demographic, and occupational variables on the level of job adjustment. The enter type was used to enter variables in the SPSS main window. In this method, all independent variables are entered concurrently into the model to determine the effect of all significant and nonsignificant variables on the dependent variable.

The model's R^2^ value was 0.48. In this regard, the desire to remain in the position of healthcare midwife was the most accurate predictor of job adjustment (*p* < 0.000). The proportion of income to expenses (*p* < 0.05), the structure of the midwife's working environment in comprehensive rural health service centers or healthcare units (*p* = 0.021 and *p* = 0.009, respectively), and the presence or absence of severe work problems during the period of employment in this profession (*p* = 0.008) were among the significant predictors of job adjustment in the healthcare role. Moreover, job satisfaction and organizational commitment were identified as strong predictors of job adjustment for healthcare midwives (*p* = 0.013 and *p* < 0.001, respectively).

This model had no significant predictive relationship between job adjustment and age, covered population, interest in the profession, economic class, housing price assessment, type of employment, organizational position, or previous job (Table 4).

**Table 4:** Evaluation of the effect of individual and job variables on the level of job adjustment using multiple linear regression model in healthcare midwives participating in the study in West Azarbaijan province in 2022 (*n* = 310)

Variable	B	SE	β	Confidence level		*P*-value
				**Min**	**Max**	
**Desire to stay in the profession**						
No desire (**reference**)						
Very low	1.839	2.616	0.048	-3.311	6.989	0.438
Low	7.709	2.809	0.194	2.180	13.238	0.006
Average	6.475	2.662	0.261	1.235	11.715	0.016
High	9.528	2.779	0.381	4.058	14.999	0.001
Very high	11.799	3.016	0.478	5.862	17.737	< 0.001
**Income adequacy**						
Very inadequate (**reference**)						
Inadequate	2.960	1.522	0.135	-0.036	5.957	0.053
Partly adequate	3.909	1.707	0.170	0.550	7.269	0.023
Adequate and very adequate	6.323	2.516	0.165	1.370	11.276	0.013
**Place of occupation**						
Urban health center (**reference**)						
Urban–rural health center	3.228	1.390	0.137	0.492	5.964	0.021
Healthcare unit	3.154	1.203	0.143	0.787	5.521	0.009
**Facing with service problems**						
Yes						
No	3.953	1.488	0.123	1.023	6.882	0.008
Job Satisfaction	0.022	0.009	0.121	0.005	0.040	0.013
Organizational Commitment	0.139	0.032	0.233	0.076	0.203	< 0.001

**Fig. 3:**
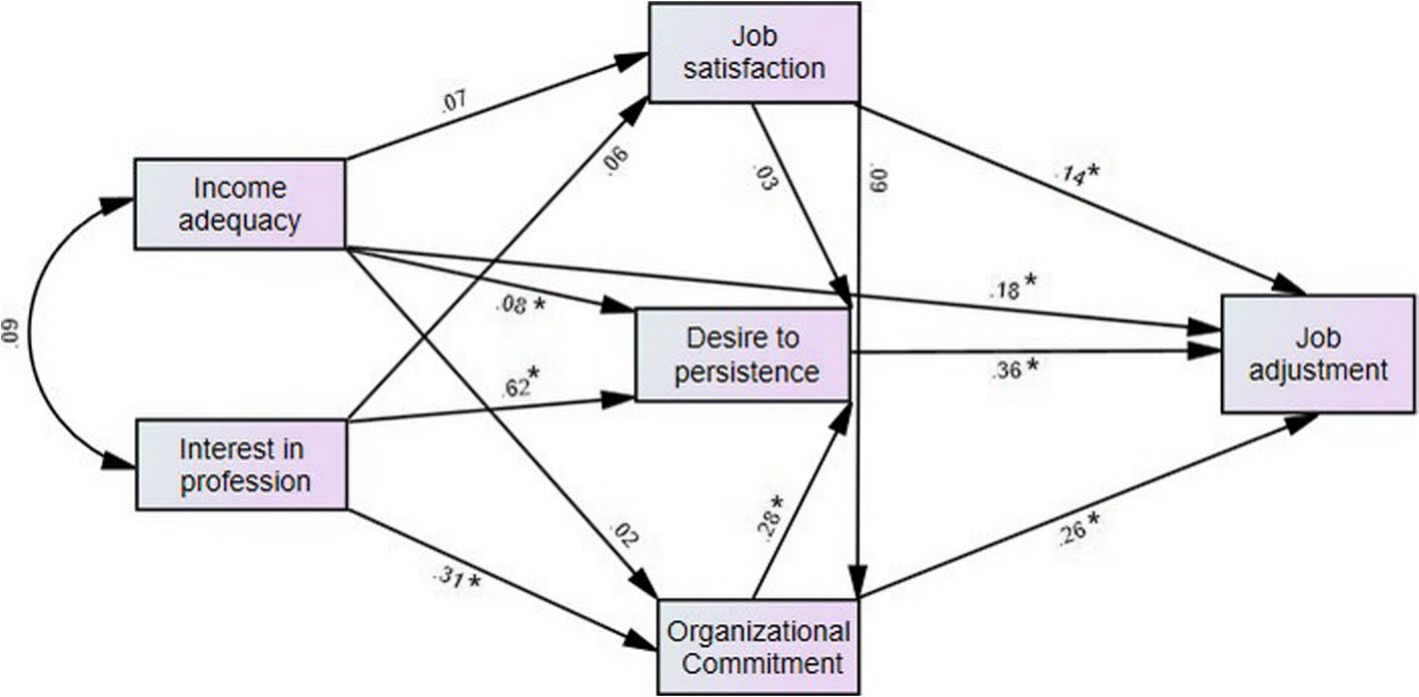
Path analysis of the factors affecting job adjustment with standard coefficients in healthcare midwives participating in the study in West Azarbaijan province in 2022 (*n* = 310), (*:*p* < 0.05)

Path analysis was used to determine the direct and indirect effects of the factors on job adjustment levels. The variables of housing price, economic class, age, and covered population were not significantly correlated with job adjustment in the model derived from regression analysis. These four variables were therefore eliminated from the path analysis diagram. The results indicated that the model's fit was satisfactory. Thus, an indirect path was drawn from adequate income to interest in the profession to obtain better-fitting indices, and a better model fit was achieved. The fit indices for the initial and final models are presented in Table 5 and Fig. 3, respectively.

**Table 5:** Fit indices of the initial and final models

**Fit index model**	Chi-square divided by degree of freedom **(CMIN/DF)**	***P***-**value**	Comparative fit index **(CFI)**	Goodness-of-fit index **(GFI)**	Root Mean Square Error of Approximation index **(RMSEA)**
Primary model	1.335	0.263	0.999	0.997	0.033
Final model	0.190	0.663	1.000	1.000	0.000

In the final model, the ratio of the chi-square statistic to the degree of freedom was less than 1; therefore, the model provided an excellent fit. When the CFI and GFI are closer to 1, the model fits better, whereas the model fit is rejected at critical values of 0.9. In this study, in the final model, the model fit was less than 0.001, indicating that the model fit was excellent. While values of < 0.08 are acceptable for the RMSEA, values of < 0.05 are preferable. This value was 0.001 in the final model, indicating that the final model exhibited a very good fit.

The direct path of the effect of the variables of adequate income to expenses, organizational commitment, desire to remain in the profession, and job satisfaction is statistically significant (*p* < 0.05), according to the results of the table. The relationship between income and expenses, interest in the profession, and organizational commitment in drawing paths significantly impacted the desire to remain in the profession. However, the variable of desire to remain in the profession had the greatest effect on job adjustment (β = 0.361), and interest in the midwifery profession had the greatest direct effect on the desire to remain in the healthcare midwifery profession (β = 0.617). (Table 6).

**Table 6:** Standard and non-standard path coefficients of the path analysis model of the variables affecting job adjustment in healthcare midwives participating in the study in West Azarbaijan province in 2022 (*n* = 310)

Variable	B	β	SE	*P*-value
Adequacy of income to expenses → job adjustment	2.969	0.179	0.749	< 0.001
Desire to stay → job compatibility	3.447	0.361	0.491	< 0.001
Organizational commitment → job adjustment	0.184	0.264	0.035	< 0.001
Job satisfaction → job adjustment	0.166	0.137	0.054	0.002
Adequacy of income to expenses → job satisfaction	0.960	0.070	0.782	0.219
Interest in profession → job satisfaction	0.594	0.065	0.523	0.256
Adequacy of income to expenses → organizational commitment	0.447	0.019	1.287	0.728
Interest in the profession → organizational commitment	4.957	0.311	0.860	< 0.001
Job satisfaction → organizational commitment	0.151	0.087	0.093	0.105
Adequacy of income to expenses → desire to stay	0.144	0.083	0.064	0.024
Interest in the profession → desire to stay	0.717	0.617	0.045	< 0.001
Organizational commitment → desire to stay	0.020	0.277	0.003	< 0.001
Job satisfaction → desire to stay	0.005	0.036	0.005	0.331

According to the results of the path analysis, interest in the profession had an indirect effect on job adjustment via job satisfaction, desire to remain in the profession, and organizational commitment. The results indicated that an increase of one unit in interest in a profession increased job adjustment by 34% when compiling the direct and indirect effects. The variable of desire to remain in the profession directly affected job adjustment, with a 36% increase per unit increase in the variable of desire to remain in the profession. The ratio of income to expenses influenced job adjustment directly and indirectly through job satisfaction, organizational commitment, and desire to remain in the profession.

In general, an increase of one unit in income sufficiency increased job adjustment by 22%. Job satisfaction had both direct and indirect effects on job satisfaction by influencing organizational commitment and desire to remain in the profession; therefore, a one-unit increase in job satisfaction and desire to remain in the profession increased job satisfaction by 18%. Organizational commitment had a significant direct effect on job adjustment and an indirect effect on job adjustment via the desire to remain in the profession. Since higher scores indicate greater organizational commitment, increased organizational commitment may increase one's desire to remain in the profession and, as a result, job adjustment. In addition, an increase of one unit in organizational commitment increased job adjustment by 36% via the mediating effect of the desire to remain in the profession.

Generally, organizational commitment and desire to remain in the profession impacted job adjustment most among the study variables. Job satisfaction, interest in midwifery, and adequate income were significant factors influencing job adjustment (Table 7).

**Table 7:** Direct, indirect and general effects of factors affecting job adjustment in healthcare midwives participating in the study in West Azarbaijan province in 2022 (*n* = 310)

Variable	Direct effect	Indirect effect	Total effect
Interest in the profession	-	0.347	0.347
Desire to stay	0.361	-	0.361
Adequacy of income to expenses	0.179	0.049	0.228
Job satisfaction	0.137	0.045	0.182
Organizational commitment	0.264	0.100	0.364

## Discussion

According to the study's findings, more than half of the healthcare midwives experienced moderate levels of job adjustment. The greater the organizational commitments and job satisfaction, the greater the job adjustment of healthcare midwives; consequently, these variables may be considered significant predictors of job adjustment. The desire to remain in this profession was also one of the significant factors related to the increase in job satisfaction among midwives, which could aid in their job adjustment to some degree.

According to studies, various factors, such as job adjustment, organizational trust, job satisfaction, and organizational commitment, interact [21]. Organizational commitment was directly and strongly affected by job satisfaction. Increased job satisfaction prompted employees to feel a greater sense of responsibility for the organization, resulting in enhanced productivity and personal fulfillment. It fostered a constructive and pragmatic outlook on organizational commitment, its guiding principles, and the desire to remain within the organization [22]. Another Iranian study found a direct correlation between all domains of organizational commitment and job satisfaction, and job satisfaction was suggested as a predictor of organizational commitment [40].

Despite being dissatisfied with their salaries, midwives remain in Iran's health reform plan because they cannot find a higher-paying job per professional skills; they have a strong desire to work based on and make use of their acquired abilities, have a strong sense of social utility, and are considered to tolerate a lighter burden of responsibility and far fewer job pressures than their peers in maternity hospitals and tertiary hospital wards [41].

According to our knowledge, no previous research in Iran has examined the job adjustment status of midwives in the role of a healthcare provider or the factors influencing this job adjustment. In most Middle Eastern and North African countries, the absence of independent professional associations and the promotion of midwifery management pose significant obstacles for midwives. A study conducted in Jordan revealed that the doctor-dominated system had neglected the professional identity of midwives within the health care system. Midwifery has no place in primary health strategies, and midwives cannot perform their roles and responsibilities to the fullest extent. This decreased the quality of midwifery services, the midwives' motivation, desire to remain in the profession, and the ability to work together continuously [42].

In various studies, the reasons for midwives' inability to adapt to the conditions of the work environment and, as a result, reduce the quality of midwifery services, the lack of sufficient supervision on the division of midwifery duties and the high workload of midwives, the gap in the basic knowledge of midwifery, the lack of appropriate management and evaluation standards [43], poor working conditions and a lack of in-depth knowledge [44], and a lack of midwifery education have been cited as contributing factors [45]. In the present study, among the domains of job adjustment, the value of altruism received the highest score for improving job adjustment. In contrast, the value of autonomy received the lowest score.

In the Netherlands, career advancement, social support at work, and professional independence were identified as significant predictors of job satisfaction; facilitating the career advancement of midwives was regarded as one of the most pressing issues for healthcare providers in this country [46]. In line with these studies, a Mashhad study examined the experiences of employed midwives in the family physician program. Professional conflicts caused by a lack of professional independence and ambiguity in the role of healthcare providers were identified as one of the program's most significant obstacles [47]. Multiple responsibilities in a short period of time, non-compliance with the basic training of healthcare midwives, sub-optimal management and supervision, and insufficient ongoing training were some of the reasons for these findings [48].

In Iran, care is frequently physician-centered, but a lack of professional autonomy and a lack of professional authority confound their careers. Nonetheless, in the theoretical and clinical training of midwifery students, the central role of midwives as the supporter of mothers in promoting the health of women and fetuses/newborns is emphasized, and this sense of duty and compassion enhances the altruistic value of midwives [49].

In this study, external factors (health factors) were the most influential on job satisfaction; therefore, salary and job security were the most important, and working conditions were the least. A study of health system personnel in Cambodia revealed that financial incentives and equitable payment mechanisms affect employee job motivation [50]. Improved employment laws for midwives, particularly regarding salaries and benefits, improved job security, merit pay and fair payment, and the use of midwives in management positions can increase their job satisfaction and facilitate the delivery of high-quality care [51]. According to the participants, job responsibility was the most significant internal factor, while work itself and job position were the least significant (motivational factors).

A study examining the motivational factors of Iranian female employees revealed a correlation between reward satisfaction and the desire to attain managerial positions [52]. Their satisfaction can be increased by selecting senior and middle managers from midwifery professionals, considering their job competence, and strengthening the midwifery management structure at ministerial levels. According to research conducted in Sri Lanka, the placement of midwives in organizational positions commensurate with their academic credentials and the assurance of job security are directly related to job satisfaction [53].

A further finding of this study was the high level of organizational commitment among healthcare midwives, with emotional commitment being the highest, indicating an interest in and a sense of belonging to the midwifery profession. According to Allen and Meyer, the relationship between organizational emotional commitment and variables such as group cohesion, organizational atmosphere, and equitable distribution of resources is more significant than the relationship between organizational emotional commitment and continuance commitments [54]. According to a study conducted in Malaysia, job position, increased autonomy and authority in decision-making, and ease of interaction with coworkers were significant predictors of organizational commitment and job satisfaction [55].

According to the study's findings, organizational factors had a greater impact on the job adjustment of these healthcare providers than demographic variables. Existing research indicates that variables such as age and income are directly related to job adjustment [56–58]. Age appears to be associated with an increase in fatigue and burnout, and the accumulation of stressors over time reduces midwives' job satisfaction and adjustment. In this study, midwives who experienced difficulties adjusting to their jobs had poorer adjustment. Job problems, the failure to implement the methods and principles of employee career counseling, and insufficient interactive skills training in the workplace can gradually erode self-efficacy and lead to job burnout, diminishing job satisfaction and, consequently, the ability to adapt to new tasks. According to a study conducted in Ethiopia, marriage is the strongest predictor of happiness in this regard. The lives and behaviors of married midwives are more stable, and they receive more encouragement and support from their partners [59].

The analysis of the relationships between workplace structure, facing job problems, income adequacy, desire to remain in the profession, job satisfaction, and organizational commitment with job adjustment revealed that organizational commitment and desire to remain in the healthcare profession had the greatest effect on job adjustment. Still, interest in the profession, income adequacy, and job satisfaction also had a significant direct and indirect effect. Some factors, including respect for dignity and job status, as well as the level of income, can be considered crucial policymakers' strategies for enhancing the satisfaction of healthcare workers [60].

On the other hand, less workload and interest in the profession [61] and a safe organizational environment substantially impact the adjustment to working conditions and retention of healthcare midwives [62]. Lack of stability and job security, irregular payments, insufficient supervision and lack of job support [63], and per capita payment were factors that contributed to job abandonment [46]. Improved financial and non-financial incentives, supportive supervision, guaranteed job security, and reformed job structure of midwives working in health centers to achieve the Millennium Development Goals (MDGs) and sustainable development are regarded as effective strategies to increase motivation and discourage health workers from quitting their jobs.

As with other cross-sectional studies, this study had limitations. Although valid and reliable standard instruments were used to measure the level of job satisfaction and adjustment of the participants, the relative understanding of these variables is frequently based on the participants' subjective perceptions of the response situations during the study. In addition, caution and job considerations are not always ineffective in the participants' responses, even when the questionnaire is completed online and anonymously. Furthermore, regulations and different organizational conditions among the study's respondents led to a variety of responses, which may affect the generalizability of these results to their peers in other provinces of the country with different cultural, social, and economic characteristics; consequently, the results should be interpreted with greater caution.

## Conclusion

Our findings suggest that healthcare planners and policymakers should concentrate their efforts and policymaking on incentivizing healthcare midwives to remain in their profession, which may result in greater work adaptation. By increasing the midwives' job satisfaction and organizational commitment, it is possible to improve the quality of the services provided.

It is recommended that future researchers conduct a more in-depth and multifaceted investigation of the factors that improve or impede adjustment to the healthcare provider's role using qualitative studies and other methods. It is hoped that the obtained results will be used to remove obstacles and some challenges of the new healthcare program, facilitate the adjustment of midwives to the responsibilities of their profession, and inform planners of healthcare systems about evidence-based planning and the neglected and unstated challenges faced by midwives in this position.

## Data Availability

The datasets used and/or analyzed during the current study are available from the corresponding author on reasonable request.

## Abbreviations

AGFI: Adjusted Goodness-of-Fit Index
CFI: The Comparative Fit Index
GFI: Goodness of Fit Index
ICC: Intraclass Correlation Coefficient
RMSEA: The Root Mean Square Error of Approximation
SEM: Structural Equation Modelling
